# Supplementary material for: Direct impact of COVID-19 by estimating disability-adjusted life years at national level in France in 2020
Source: PLoS One. 2023 Jan 24;18(1):e0280990. doi: 10.1371/journal.pone.0280990 (PMC9873186; doi:10.1371/journal.pone.0280990)
Supplement: S1 Table — (DOCX) [file pone.0280990.s001.docx]

**S1 Table: Duration of hospitalization and hospitalization with intensive care in 2020**

| **1.1** | **Mean duration of hospitalization in 2020** | | | |
| --- | --- | --- | --- | --- |
| S/No | Age group (years) | Mean duration (days) | Min 95%CI | Max 95%CI |
| 1 | 0 – 9 | 5.048 | 0.495 | 17.683 |
| 2 | 10 -19 | 6.484 | 0.553 | 24.851 |
| 3 | 20 – 29 | 6.725 | 0.702 | 23.836 |
| 4 | 30 -39 | 7.849 | 0.791 | 28.392 |
| 5 | 40 – 49 | 10.168 | 0.911 | 39.337 |
| 6 | 50 – 59 | 12.522 | 1.068 | 49.829 |
| 7 | 60 – 69 | 15.961 | 1.264 | 67.582 |
| 8 | 70 – 79 | 18.612 | 1.476 | 82.148 |
| 9 | 80 – 89 | 19.544 | 1.652 | 88.044 |
| 10 | 90 + | 19.578 | 1.550 | 91.735 |
| **Total** | **All** | **12.249** | **1.0462** | **51. 3437** |
| **1.2** | **Mean duration of hospitalization with intensive care in 2020** | | | |
| 1 | 0 – 9 | 10.895 | 1.011 | 42.368 |
| 2 | 10 -19 | 13.630 | 1.029 | 56.832 |
| 3 | 20 – 29 | 15.554 | 1.133 | 66.233 |
| 4 | 30 -39 | 18.392 | 1.618 | 76.065 |
| 5 | 40 – 49 | 21.977 | 2.085 | 90.051 |
| 6 | 50 – 59 | 26.392 | 2.392 | 113.265 |
| 7 | 60 – 69 | 28.687 | 2.606 | 127.358 |
| 8 | 70 – 79 | 28.790 | 2.375 | 134.726 |
| 9 | 80 – 89 | 20.139 | 1.472 | 96.063 |
| 10 | 90 + | 17.990 | 1.103 | 88.136 |
| **Total** | **All** | **20.2446** | **1.6824** | **89.1097** |
