# Supplementary material for: Direct impact of COVID-19 by estimating disability-adjusted life years at national level in France in 2020
Source: PLoS One. 2023 Jan 24;18(1):e0280990. doi: 10.1371/journal.pone.0280990 (PMC9873186; doi:10.1371/journal.pone.0280990)
Supplement: S2 Table — (DOCX) [file pone.0280990.s002.docx]

**S2 Table: Scenario analyses highlighting the impact of number of infections and durations on YLD and DALYs, by sex in France, 2020**

| **Health states** | **Parameters** | **YLD (UI 95%)** | **DALY (UI 95%)** |
| --- | --- | --- | --- |
| **Acute symptomatic COVID-19 infections** | | |  |
| Female | Reference | 1984 [1983 – 1985] | 424 731 [424 730 – 424 732] |
| Male | Reference | 2225 [2224 – 2256] | 562 009 [560 008 – 562 010] |
| Female | Increased mild/moderate cases by 50% | 2582 [2580 – 2583] | 425 329 [425 328 – 425 331] |
|  | Increased mild/moderate cases by 75% | 2881 [2880 – 2883] | 425 629 [425 627 – 425 630] |
| Males | Increased mild/moderate cases 50% | 2713 [2712 – 2714] | 562 498 [562 496 – 562 499] |
|  | Increased mild/moderate cases 75% | 2957 [2956 – 2959] | 562 742 [562 740 – 562 743] |
| Female | Duration of 7 days | 1584 [1584 – 1585] | 424 332 [424 331 – 424 332] |
|  | Duration of 14 days | 2382 [2381 – 2384] | 425 130 [425 129 – 425 131] |
| Male | Duration of 7 days | 1899 [1899 – 1900] | 561 683 [561 683 – 561 684] |
|  | Duration of 14 days | 2550 [2549 – 2551] | 562 334 [562 234 – 562 336] |
| **Post-acute consequences of COVID-19/Long COVID** | | |  |
| Female | Duration of 28 days (Reference) | 2147 [2146 – 2148] | 424 894 [424 893 – 424 896] |
| Male | Duration of 28 days (Reference) | 1824 [1823 – 1825] | 561 608 [561 607 – 561 610] |
| Female | Duration of 84 days | 6173 [6169 – 6177] | 428 920 [428 916 – 428 924] |
| Male | Duration of 84 days | 5244 [5241 – 5248] | 565 028 [565 025 – 565 032] |
| Female | Duration of 140 days | 10198 [10192 -10 205] | 432 946 [432 939-432 952] |
| Male | Duration of 140 days | 8664 [8658-8670] | 568 448 [568 443- 568 454] |
