# Supplementary material for: Direct impact of COVID-19 by estimating disability-adjusted life years at national level in France in 2020
Source: PLoS One. 2023 Jan 24;18(1):e0280990. doi: 10.1371/journal.pone.0280990 (PMC9873186; doi:10.1371/journal.pone.0280990)
Supplement: S3 Table — (DOCX) [file pone.0280990.s003.docx]

|  |  |  |  |  |  |  |  |
| --- | --- | --- | --- | --- | --- | --- | --- |
|  | **Male** |  |  |  | **Female** |  |  |
| **Age group** | **Mild/Moderate** | **Severe** | **Critical** | **Age group** | **Mild/Moderate** | **Severe** | **Critical** |
| 00-09 | 17 | 1 | 2 | 00-09 | 16 | 1 | 1 |
| 10-19 | 101 | 1 | 2 | 10-19 | 112 | 2 | 2 |
| 20-29 | 193 | 3 | 5 | 20-29 | 239 | 6 | 4 |
| 30-39 | 168 | 6 | 16 | 30-39 | 214 | 9 | 11 |
| 40-49 | 157 | 17 | 45 | 40-49 | 198 | 12 | 20 |
| 50-59 | 146 | 39 | 120 | 50-59 | 176 | 24 | 55 |
| 60-69 | 94 | 77 | 248 | 60-69 | 106 | 48 | 108 |
| 70-79 | 58 | 119 | 288 | 70-79 | 62 | 79 | 125 |
| 80-89 | 33 | 126 | 81 | 80-89 | 48 | 138 | 51 |
| 90+ | 10 | 45 | 8 | 90+ | 26 | 83 | 8 |
|  | **977** | **433** | **814** |  | **1197** | **402** | **385** |
|  |  |  |  |  |  |  |  |

**S3 Table : Years of Life Lived due to acute symptomatic COVID-19 infections in France in 2020 by age, sex and severity level**
