# Supplementary material for: Direct impact of COVID-19 by estimating disability-adjusted life years at national level in France in 2020
Source: PLoS One. 2023 Jan 24;18(1):e0280990. doi: 10.1371/journal.pone.0280990 (PMC9873186; doi:10.1371/journal.pone.0280990)
Supplement: S4 Table — (DOCX) [file pone.0280990.s004.docx]

**S3 Table: Comparison of COVID-19 DALYs studies in European Countries**

| **S/No** | **Country and period of analysis** | **Life expectancy table** | **Mortality** | **Morbidity** | **YLL** | **YLD** | **Post-acute consequences of COVID-19** | **DALYs/100 000** | **%**  **YLD** |
| --- | --- | --- | --- | --- | --- | --- | --- | --- | --- |
| 1 | Germany  [2020] | National life tables-2016/2018 | 31 638 | 1 717 006 | 303 608 | 2033 | No | 542 | 0.7% |
| 2 | Scotland  [2020] | GBD-2019 | 6167-6845 | 641 789 | 94 633-106 357 | 1886 | Yes | 1770-1980 | 2% |
| 3 | Malta  [7 Mar. 2020 - 31 Mar.2021] | GBD-2019 | 331 | 70 421 | 5229 | 250 | Yes | 1086 | 5% |
| 4 | The Netherlands  [2020] | GBD-2019 | 19 980 | 928 476 | 271 900 | 1600 | No | 1570 | 1% |
| 5 | France [2020] | GBD-2019 | 72 735 | 1 585 032 | 982 531 | 8179 | Yes | 1472 | 1% |
| 6 | Republic of Ireland [Mar. 2020 - Mar. 2021] | GBD-2019 | 4500 | 220 273 | 50 823 | 800 | Yes | 3798 | 1.5% |
| 7 | Denmark [26 Feb. 2020 - 25 Feb.2021] | GBD-2019 | 2383 | 211 823 | 29 689 | 492 | Yes | 520 | 1% |
