# Supplementary material for: Direct impact of COVID-19 by estimating disability-adjusted life years at national level in France in 2020
Source: PLoS One. 2023 Jan 24;18(1):e0280990. doi: 10.1371/journal.pone.0280990 (PMC9873186; doi:10.1371/journal.pone.0280990)
Supplement: Attachment — Submitted filename: Response to reviwers_05072022.docx [file pone.0280990.s005.docx]

Paris, July 5, 2022

Subject: Major revisions for “PONE-D-22-11149: Direct impact of COVID-19 by estimating disability-adjusted life years at national level in France in 2020”

Dear Dr. Mossong,

We would like to thank you for considering our manuscript. Both reviewers’ gave useful comments that have improved the quality of our manuscript.

We have answered the reviewer’s comments and modified the manuscript accordingly. There are two manuscript files (one with track changes and one clean version). The line numbers in the comment file correspond to the clean version file.

We have updated competing statement as follows:

"I have read the journal's policy and the authors of this manuscript have the following competing interests: Romana Haneef is the first and corresponding author of this paper, and is the section editor of “health information system” of “Archives of Public Health”. Brecht Devleesschauwer is one of the co-authors of this paper, and is academic editor at PLOS ONE and the guest editor of the article collection on “burden of disease” of “Archives of Public Health”. All other authors declare that they have no competing interests related to the work. This does not alter our adherence to PLOS ONE policies on sharing data and materials."

We would be grateful for your kind support for publication of this manuscript.

Yours sincerely,

Romana Haneef

**Journal Requirements:**

When submitting your revision, we need you to address these additional requirements.

1. Please ensure that your manuscript meets PLOS ONE's style requirements, including those for file naming. The PLOS ONE style templates can be found at

<https://journals.plos.org/plosone/s/file?id=wjVg/PLOSOne_formatting_sample_main_body.pdf> and <https://journals.plos.org/plosone/s/file?id=ba62/PLOSOne_formatting_sample_title_authors_affiliations.pdf>

**Response:** Thank you. We have removed the physical address of the corresponding author from the title page and ensured that manuscript meets the PLOS ONE’s style requirements.

2. Please clarify within the ethics statement whether the current study had authorization of the CNIL for the treatment of personal health data.

**Response:** We had mentioned this in the main text of the manuscript (line 211) and have also clarified it within the ethics statement as follows:

“The study was based on aggregated and anonymous data from national mandatory database (SIVIC: article L3131-9-1 and R3131-10-1 code de la santé publique; SIDEP: article 11 loi n°2020-546 and article 8 and following décret n°2020-551). Furthermore, Santé publique France has been granted by French Law specific access to these national databases in order to carry out its mission of health monitoring and response to health crisis (article L. 1413-7 code de la santé publique). Therefore, no ethics approval or consent of participant was needed, as the processing made by Santé publique France is provided for by the Law.”

3. Thank you for stating the following in the Competing Interests section: 
"I have read the journal's policy and the authors of this manuscript have the following competing interests: Romana Haneef is the first and corresponding author of this paper, is the section editor of “health information system” of “Archives of Public Health”. Brecht Devleesschauwer is the co-authors of this paper, is the editor of article collection on “burden of disease” of “Archives of Public Health”. All other authors declare that they have no competing interests related to the work."

Please confirm that this does not alter your adherence to all PLOS ONE policies on sharing data and materials, by including the following statement: "This does not alter our adherence to PLOS ONE policies on sharing data and materials.” (as detailed online in our guide for authors <http://journals.plos.org/plosone/s/competing-interests>).  If there are restrictions on sharing of data and/or materials, please state these. Please note that we cannot proceed with consideration of your article until this information has been declared.

Please include your updated Competing Interests statement in your cover letter; we will change the online submission form on your behalf.

**Response:** We have added the updated Competing Interests statement in the cover letter.

**Response to reviewers’ comments**

**Reviewer #1:**

General remark: the article is written in Standard English as required but would nevertheless benefit from a professional proof reading.

**Response:** Thank you very much. While we chose not to hire professional proof reading services, we made a thorough language check and revision of the manuscript.

General remark: DALY are mainly a comparative measure. While the present paper presents the 1st analysis of the burden of C-19 in France it remains limited to the comparison by age and gender. It shows that the differences by sex are striking and the concentration of disease burden in men already in middle age is very noticeable. This is an important aspect of the epidemiology of C-19, which has practical and political implications, and illustrates the benefit of burden of disease indicators compared to classical epidemiological measures. However, in the paper more attention could be paid to these aspects by highlighting these differences in the results section and in the discussion. So far, the scenario analyses (as a methodological by-product) as well as the absolute DALY, which in themselves are not very intuitive, take up comparatively large space.

**Response:** Thank you very much for this comment. Indeed, we agree with you and we compared these estimates with other countries. Accordingly, we have added the following text in the result and discussion sections:

*Results (line 265-267)*

*« The YLL among people [….] (Fig 1) and women accounted a higher share of YLL than men (37% vs 31%). On the other hand, among people younger than 70, men accounted for a much higher share of YLL than women (31% vs 19%). »*

*Line 277*

*« Contrary to YLL, among people younger than 70, women accounted for a slightly higher share of YLD than men (69% vs 66%). »*

*Discussion (line 433)*

*« Among people younger than 70, YLL were much higher among men than women and these results have important implications for improving health care and prevention. In general, these results highlight the important differences by sex. The concentration of disease burden among men already in middle age is very noticeable. This is an important aspect of the epidemiology of COVID-19, which has practical and political implications, and emphasizes the benefit of burden of disease indicators compared to classical epidemiological measures. »*

In detail:
Line 70: It should be „November 2020” not “2021”

**Response:** This has been updated in the manuscript (line 67).

*« November 2020 »*

Line 117: Please state explicitly the case definition for the present analysis not only what is in the data: were all positive cases (PCR and rapid tests) included. Was a confirmatory 2nd rapid test required? Were there any exclusions defined (e.g. based on plausibility checks)?

**Response:**  For this study, only positive cases with PCR or rapid/antigen tests were included. The same case definition as SI-DEP was used to identify the positive cases with PCR and rapid/antigen tests. If the first rapid/antigen test was positive and followed by a negative confirmatory PCR test, it was still considered as a positive case.

We have added the following case definition for the present analysis and updated it in the manuscript file (Line 114):

*« For this study, we considered all the cases who were tested positive (with PCR or antigen tests) once during a period of 60 days and were included in the present analysis. The case definition of positive cases was based on the SI-DEP database as 60 days […]. »*

Line 118: what is meant by “infra-national”

**Response:** We meant sub-national and is replaced by « infra-national » in the manuscript (Line 113).

*« Subnational »*

Lines 127/128: The whole concept of uncertainty should be explained in more detail. If one assumes a complete collection of data, there actually no uncertainty would be expected. Under what assumptions and with what methods was uncertainty calculated. The explanation that is given only refers to uncertainty intervals for YLD. Do uncertainty intervals for DALY only refer to the uncertainty in YLD? Is there no uncertainty assumed for YLL? If a concept of uncertainty is applied it should be said which aspects it covers and which aspects not.

**Response:** Thank you very much for this comment. We have added an explanation under the section of « scenario analyses » (line 220-238) as follows and have deleted the sentence (line 125) to avoid confusion on uncertainty:

*«We performed a sensitivity analysis that corresponds to the variation of input parameters including the number of mild/moderate cases and the duration according to the scenario.*

*During the epidemic, many cases with mild and moderate symptoms may have remained undiagnosed/untested and not registered in the SI-DEP database. At the time of submission of this study, the true incidence of COVID-19 for the whole year of 2020 in France was not available. Therefore, to evaluate the impact of underreporting underestimation of the burden of disease, we varied the proportion of reported cases by increasing them by 50% and 75%, respectively.*

*The duration of infection for a mild/moderate case was not recorded in SI-DEP. The transmission of the virus is different across the whole population, influenced by environmental and behavioral factors, and varied from person to person. Therefore, the duration of mild/moderate cases remains uncertain. To take into account the uncertainty around this parameter, we varied the duration from 7 days to 14 days. Similarly, the duration of post-acute consequences of COVID-19 was based on transition probabilities calculated in a previous study. At the time this study was performed, no data on duration were available.*

*Therefore, we varied the number of mild/moderate cases and the duration to quantify the uncertainty intervals of YLD as described below. We did not apply this concept to the YLL as the national mortality database (i.e., CépiDc) is considered a robust data source with good quality of data and no data adjustment was applied. »*

Lines 140/141: It would be interesting to show the resulting severity distribution, maybe even by time and/or by age/sex.

**Response:** As suggested, we have mentioned in the method section and added a figure with a brief description in the results section as follows:

*Method (health states) (line 139)*

*« We also described the severity distribution of these health states by age and sex. »*

*Results (line 283)*

*« The severity distribution of YLD varied among men and women (Fig 3). Overall, the proportion of YLD due to mild/moderate cases was 60% among women whereas 44% among men. Among women younger than 70, they shared a larger proportion of YLD due to mild/moderate cases than women aged 70 and above (89% vs 11%). The proportion of YLD due to severe cases was almost similar in both sexes (20% women vs 19% men). The YLD due to critical cases was higher among men (37%) than women (20%). »*

*Fig 3 (line 290): Years of Life Lived due to acute symptomatic COVID-19 infections in France in 2020 by age, sex and severity level »*

Lines 160/161: The Australian study as well calculates durations from hospital data and ends up with somewhat different results compared to the French study (<https://www.aihw.gov.au/getmedia/a69ee08a-857f-412b-b617-a29acb66a475/aihw-phe-287.pdf.aspx?inline=true>). On the other hand, the Australian results compare quite well with the assumptions on durations made in the German study. Even if the impact on YLD is limited such aspects could be discussed in more detail.

**Response:** Thank you for this comment. We have cited the « Australian study » in the list of countries who calculated the DALYs estimates under the discussion section (line 341):

*« Other than European countries, Australia has also calculated DALYs estimates using the Burden of Disease consensus disease model for COVID -19 (AIHW). »*

The following text on different assumptions for duration in relation to the results has been added under the discussion section (line 347):

*« For example, Australia applied the same assumption for the duration of hospitalized and critical cases (i.e., average length of stay) as France but had different results. The hospital stay among people aged 80 and over was longer in France than in Australia (i.e., 20 days vs 12 days). These differences highlight that patients were critically ill and were required longer time in intensive care in France than in Australia. The heterogeneity in health care in the hospitals and health insurance system might affect the length of stay in the hospital, consequently, which can influence the YLD estimates. Overall, the length of stay in hospital increased with age in both countries. »*

Lines 177 et seq.: Please be a bit more explicit and detailed again on what definitions exist in the data, which case definitions were made for the analysis and possible exclusions due to data cleaning. Generally, there are three potential types of C-19 deaths: (i) dying with C-19 infection (but maybe has died in a car crash) (ii) dying due to C-19, where C-19 is one of several causes on the death certificate and (iii) deaths with C-19 as the main underlying cause of death. What definitions have been received here.

**Response:** Precisely, we included those deaths with COVID-19 as the main underlying causes of death in our analysis. This was already reported in the text under the method section and we updated it more explicitly as requested:

*Method (line 187)*

*« The underlying causes of death are selected using an automatic system (IRIS software [21]) for coding and prioritizing multiple cause of death. »*

*Line 197*

*« COVID-19 deaths were defined as deaths reported with COVID-19 as the main underlying cause of death according to the WHO recommendations. »*

*Line 201*

***«*** *In the main analysis, we included those deaths with COVID-19 as the main underlying cause of death. »*

As I understand it, the definition in the 80% and the 20% may not be quite the same? A time lag in the data is also mentioned here.

**Response:** We already mentioned in the manuscript that the 80% deaths are based on underlying cause of death and 20% include deaths reported as the free text with a mention of COVID-19 (line 192 – 195). The COVID-19 death is the common word and the algorithm applied to extract COVID-19 terms from the free-text section of the death certificate, has a high performance of 93%.

How do the figures on deaths used here compare with those that were published very timely during the pandemic and reported to the WHO, for example? Are there major differences?

**Response:** For this study, we did not formally compare this data with the WHO reported ones. It is likely that the mortality data used in the study was provided by CépiDC, are more reliable than those published by WHO, which might have counted the COVID-19 death numbers as cumulative number of deaths from all causes over time. Nevertheless, we compared mortality estimates with other countries who had performed DALYs estimations. We have added this point as further research in the discussion section as follows:

*Discussion (line 463)*

*« We recommend further research to compare the mortality estimates by the CépiDC with the WHO reported ones that were published very timely during the pandemic. »*

Lines 208-210: What is raised in the methods section should be presented under results. These aspects first come up in the discussion. Delete this in the methods section but as well briefly discuss the limited comparability of country specific analyses with GBD data.

**Response:** As requested, we have deleted this from the method section and mentioned following text in the discussion section (line 443):

*« Nevertheless, the country specific analyses had limited comparability with the GBD data due to several reasons including the use of local data sources, different case definitions, methodological choices, etc. ».*

Lines 248/249: Please stress a little bit the sex/gender differences here. Younger men account for a much higher share of YLL than younger women. This is an interesting phenomenon with many potential implications for prevention and health care.

**Response:** Thank you for this comment. We have added the following text in the result and discussion sections based on your first comment:

*Results (line 265-267)*

*« The YLL among people [….] (Fig 1) and women accounted a higher share of YLL than men (37% vs 31%). On the other hand, among people younger than 70, men accounted for a much higher share of YLL than women (31% vs 19%). »*

*(Line 277)*

*« Contrary to YLL, among people younger than 70, women accounted for a slightly higher share of YLD than men (69% vs 66%). »*

*Discussion (line 433)*

*« Among people younger than 70, YLL was much higher among men than women and these results have important implications for improving health care and prevention. In general, these results highlight the important differences by sex. The concentration of disease burden among men already in middle age is very noticeable. This is an important aspect of the epidemiology of COVID-19, which has practical and political implications, and emphasizes the benefit of burden of disease indicators compared to classical epidemiological measures. »*

Lines 272/273: It’s the other way round isn’t it? The share of acute infections within the age specific burden of C-19 is higher in younger age groups.

**Response:** Indeed, the share of YLD due to acute infections within the age specific burden of COVID-19 is higher in younger age groups (67% vs 33%) [line 276-277] but for DALYs, COVID-19 burden due to acute symptomatic infections is higher among people aged 70 and above then among people under 70 years (74% vs 26%) [line 302].

Line 278 et seq.: When presenting the scenario results you could give some information to what extend the share of YLD (in %) changes when introducing different scenarios (from 99% in the main analysis). This would be a bit more intuitive than just comparing absolute YLD.

**Response:** Thank you for this comment to further improve the manuscript. We have added the % increase in the YLD estimates by varying different scenarios as follows under the results section of « scenario analyses »:

***Results (scenario analyses)*** (line 309 – 317)

*« First, by increasing the number of positive cases by 50 % or 75%, it led to an increase of 26% (5295) and 39% (5838) of YLD, respectively, as compared to the main results of YLD (4208). In turn, 987 827 and 988 370 DALYs were estimated, respectively, as compared to the main results of DALYs (986 740). Second, by varying the duration from 7 days to 14 days, it led to an increase of 17% for both durations with a total of 3484 and 4933 YLDs, respectively. Third, by varying the duration for post-acute consequences of COVID-19 cases to 84 days, it led to an increase of 40% with a total of 11 417 YLDs […]. A detailed analysis by sex is reported in S2 Table. »*

Line 297 et seq.: The 1st paragraph of the discussion should be a summary of the main results. Here it is focused on the scenarios. However, this is not mainly a methodological study. Scenarios could be integrated later, e.g. in the limitations paragraph.

**Response:** We agree with this comment and as requested, we have moved the text related to the scenario results from the main results to the limitation section (as limitation 3 - line 393).

Lines 303/304: This conclusion is far too strong (also in the abstract). Although this is the variable with the largest effect in the scenario analyses, the impact on overall DALYs remains nevertheless very limited.

**Response:** We have updated the tone of this sentence as follows:

*Abstract (conclusions) (line 50)*

*« Post-acute consequences of COVID-19 have a higher contribution to the YLD component of the burden than acute symptomatic infections [...] »*

*Discussion and conclusions (line 332 and 472)*

*« Our analysis highlighted that post-acute consequences of COVID-19 have a higher contribution to the YLD component of the burden than acute symptomatic infections and thus […]. »*

Lines 310-312: Germany deviates somewhat more from the consensus model compared to the other countries. The analyses were published before and did not make direct reference to the consensus model. The disease model covers somewhat different health states and did not consider Long COVID because evidence was scarce at that time.

**Response:** We agree that at the time of publication of German study, very limited evidence was available. We have deleted the relevant text and added the following text in the discussion section (line 366).

*«The German study was published very early during the pandemic. The consensus disease model covered health states of acute symptomatic COVID-19 infections and did not consider post-acute consequences of COVID-19 due to scarce evidence at that time. »*

Lines 324/325: I wonder to what extend these differences may also be due to different case definitions especially deaths. In some countries all cases dying with COVID-19 are reported, in others like Germany only cases having died due to C-19 were considered, in France, as far as I understood, only cases with C-19 as the main underlying cause were included (see commentary lines 177 et seq.). This should make a difference in total DALY that could be discussed here.

**Response:** Thank you for this interesting comment. It is important to take into account the variability in data sources and the availability of official mortality estimates. As, it has been discussed in the German study that “*Moreover, persons with COVID-19 can die from multiple causes; the recording of only the main cause of death of the list of underlying causes, as is done in Germany, is problematic (Wengler A et al, 2019 and Wengler A et la, 2020). However, future analyses based, e.g., on cause-of-death statistics for 2020 will be able to reveal whether COVID-19 was given as the official cause of death in numbers comparable to the reported fatal cases of COVID-19”*.

Nevertheless, we have added the word *« different case definitions especially for death »* (line 345) and the following sentence (line 356):

*« In German study, only deaths due to COVID-19 were considered whereas, in France, the deaths with COVID-19 as the main underlying cause were included in the main analysis. This difference in case definitions of COVID-19 death might influence the number of deaths counted that can have an impact on YLLs and then total DALYs. For example, in France YLL counted higher (982 531 YLL: death with COVID-19 as underlying cause of death) than in Germany (303 608 YLL: death due to COVID). Nevertheless, it is important to take into account that both countries have different mortality data sources and it is possible that the official estimates of the causes of death were not available in Germany at the time study was conducted and it underestimated the YLL. »*

Line 321: Given the fact that Germany has the largest population it is a bit meaningless to state that it has the most infections.

**Response:** We have deleted the related sentence in text.

**Reviewer #2:** Thank you for the opportunity to review ‘Direct impact of COVID-19 by estimating disability-adjusted life years at national level in France in 2020’. This well-written paper adds to the ongoing estimates of the relative importance of death, short-term, and long-term disability in determining total COVID-19 morbidity.

**Response:** Thank you very much for this compliment.

My major concern with this paper is the implausibly low duration of long-COVID, estimated from an analysis, which in my view does not adequately distinguish censored subjects from those with events or either of these from missing data. Symptom duration was defined, roughly, as time from diagnosis to the first one-week period where neither symptoms nor hospitalization were reported; and those subjects who reported being seriously ill and then stopped logging were excluded from the analysis. Given that fatigue and brain fog are among the most common long-COVID symptoms, it is likely that the sickest patients more-often stopped reporting. Precisely the data which we would need to draw adequate conclusions, are missing.

**Response:** Thank you for highlighting this point. We fully agree with you that precise data to draw adequate conclusions on long-COVID are missing and more evidence is needed. Currently, many studies are ongoing and in near future, their results would support to rule out various aspects of long-COVID. We have already mentioned it in the method section (line 162-165) the assumption of calculating the cases of long COVID, is based on previously calculated transition probabilities (Sudre C H et al, 2021).

We have added the following text in the method section and updated it the discussion section as well:

*Method (line 163)*

*« At the time this study was conducted, some studies were ongoing to collect the data on post-consequences of COVID-19 in France. »*

*Discussion (line 404)*

*« Currently, many studies are ongoing to collect the data on post-acute consequences and at the time this study was conducted, scarce evidence was available. »*

I would prefer if the current publication relied on studies, which actively contact patients, such as those reviewed in <https://www.nature.com/articles/s41591-021-01283-z>. These are likely to be much less biased by incomplete ascertainment, especially those such as Huang et al (cited in the current paper) with near-complete response; or Kim et al (<https://bmcinfectdis.biomedcentral.com/articles/10.1186/s12879-022-07062-6>). Even these are likely to underestimate duration somewhat, since post infectious conditions tend to relapse and remit: a patient who is recovered at the time of follow up may relapse again. This should be mentioned in the Discussion.

**Response:** Thank you very much for this important point. We have cited following studies reported in the review and have mentioned this point in the discussion section (line 409):

*« Nalbandian A. et al, has provided a comprehensive review of the current literature on post-acute COVID-19, its pathophysiology, its organ-specific sequelae and relevant considerations for the multidisciplinary care of COVID-19 survivors. Several studies have shown that […] (Carfi et al, Carvalho-Schneider et al, Chopra et al, Arnold et al, Moreno-Pérez et al, Garrigues et al). Even these studies are likely to underestimate duration somewhat, since post-acute infectious conditions tend to relapse and remit: a patient who is recovered at the time of follow up may relapse again. Therefore, these results underestimate the contribution of post-acute consequences of COVID-19 to YLD. »*

I also request that clinical manifestations other than long-COVID, such as myocarditis and psychiatric illness, be considered. These are described in Nalbandian et al, cited above. For simplicity and to avoid making the paper too long, I would be satisfied if their incidence rates were summed and the disability weights averaged to provide a single term for “all other conditions.”

**Response:** Thank you for this comment and the suggestion.

« *Summing up the incidence rates and the disability weights averaged to provide a single term for ‘all other conditions’ »,* this would be interesting*.* The main focus of this paper was to calculate the burden of COVID-19 and we briefly mentioned the symptoms of long COVID reported in published literature. We did not plan to describe the clinical manifestations other than long-COVID as it was not part of the European Burden of Disease Network consensus disease model for COVID-19. Moreover, due to lack of data and resources, we were not able to perform this analysis. Nevertheless, we mentioned this point as one of the limitations of this study (limitation 6) and also recommend further research to evaluate the impact of “all other conditions” as multimorbidity and comorbidity on DALYs estimates as follows:

*Discussion (line 417)*

*« Sixth, for this study, we did not take into account the multimorbidity and comorbidity parameters in the estimation of DALYs due to the inconsistent nature of COVID-19 in presence of different chronic conditions and lack of precise data. Moreover, this aspect is currently debated and may introduce some ethical issues whose discussion is beyond the scope of this paper. Nevertheless, we recommend further research to explore the impact of multimorbidity and comorbidity on DALYs estimates. »*

Lastly, I request an extension of the literature review, comparing the current estimates with those others have found. The current paper may be the first to consider both in-hospital morbidity and long-COVID, in addition to death (many others explicitly exclude long-COVID, e.g. <https://www.researchsquare.com/article/rs-1026794/v2>) however it estimates a much higher share of YLD than <https://www.jclinepi.com/article/S0895-4356(21)00339-5/fulltext> . Please compare your findings to those of the latter paper, as well as any others you may find that compute DALY for long-COVID.

**Response:** Thank you for this comment. We have cited the relevant articles and the following text in the discussion section (line 370):

*« Scotland reported the highest contribution of post-acute consequences to YLD (76%) followed by Malta (60%) and then France (49%). This highlights the large impact on population health. It is important to take into account what kind of post-acute consequences were considered, which might explain the heterogeneity in these results. Nevertheless, more evidence is needed to draw conclusions on its impact on the population (Maia P. Smith, 2022). »*

These issues are the only ones I have with the publication of the paper. If they are fully implemented I do not anticipate a second round of edits: the writing style does not require line edits. Please let me know if anything is unclear or I can be of further assistance.

**Response :** Thank you
