# Supplementary material for: Direct impact of COVID-19 by estimating disability-adjusted life years at national level in France in 2020
Source: PLoS One. 2023 Jan 24;18(1):e0280990. doi: 10.1371/journal.pone.0280990 (PMC9873186; doi:10.1371/journal.pone.0280990)
Supplement: Attachment — Submitted filename: Response to reviwers_07102022.docx [file pone.0280990.s006.docx]

**Response to reviewers’ comments**

**Reviewer #1:**

General remark: I would still suggest having checked the text - at least some parts of the paper, especially the newly introduced parts of the results section - by a native speaker in order to make sure that what you want to say is actually understood in the same way. Some expressions still seem to be a bit misleading to me.

**Response:** Thank you. The manuscript has been re-reviewed by a native English speaker, one of the co-authors of this manuscript, Grant Wyper (Place and Wellbeing Directorate, Public Health Scotland, Glasgow, Scotland, United Kingdom).

219 et seq The description of the scenario analyses contains some repetitions and could be shortened.

**Response:** It is updated.

265 et seq “On the other hand, among people younger than 70, men accounted for a much higher share of YLL than women (31% vs 19%).” Maybe better: “On the other hand, men younger than 70 accounted for a much higher share of YLL in men (31%) than women of the same age for the share of YLL in women (19%).”
**Response:** This has been updated as follows (line 256) :

*« On the other hand, men younger than 70 years accounted for a much higher share of YLL (31% of 559 784) than women of the same age (19% of 422 747).”*

283 et seq

While Fig 3 is a very interesting presentation of results (YLD by severity) it does not show the severity distribution as is claimed in the text. Severity distribution is defined as the distribution of diseased cases (not YLD) across the levels of severity (expressed in total numbers and/or percentages). Severity distributions could be presented in a supplementary table in order to make the data/methods more transparent.

**Response:** Thanks for this precision. Fig 3 describes the YLD across three levels of severity by age and sex. The text is updated in the method section as follows (line 134):

*« These health states represent the different severity levels considered for COVID-19 cases. »*

*« We also described the YLD estimates among males and females according to mild/moderate, severe and critical cases. » (line 175)*

We have added a supplementary table as S3 Table, describing the YLD by severity level to make data more transparent.

302 et seq

The sentence “The percentage of DALYs due to acute symptomatic infections were higher among people aged 70 and above than among people under 70 years (74% vs 26%).” is still a bit confusing. What do you mean by DALY due to acute infections? Is this YLD? If yes that it should be: “People aged 70 and above account for 74% of the total YLD” and so on. Put differently, the share of YLD in DALY should be higher in younger people compared to the elderly because only very few younger people die from C-19.

**Response :** The DALYs due to acute symptomatic infections only take into account the YLD of mild/moderate, severe and critical cases (i.e., 4208). The DALYs due to acute infections do not take into account the YLD due to long COVID (i.e., 3971). The suggested YLD interpretation has already reported in YLD result section.

We have clarified this phrase to avoid any confusion as follows (line 298-302) :

*« The percentage of DALYs due to acute symptomatic infections (i.e., including only YLD of mild/moderate, severe and critical cases [4208] and YLL estimates) were higher among men than women (57% vs 43%). People aged 70 years and above had a higher DALY estimates compared to people under 70 years (728 688 vs 258 051). (Fig 4) »*

308 et seq

In line 300 it is said: “We observed that 99% of DALYs were due to mortality (982 531 YLL) and only 1% was due to morbidity (8179 YLD).” How does this distribution changes in the scenario analyses, especially when introducing longer durations for long COVID? It is frequently argued that the high share of YLL within DALY would be strongly diminished when adequately considering long COVID.

**Response :** The contribution of mortality remains high even after introducing the long duration of COVID-19 to 140 days as follows :

DALY with 140 days duration (1 001 394 = 100%) = YLL (982 531 = 98%) + YLD (18 863 = 2%).

Therefore, we have updated this argument after considering the results of current additional sensitivity analysis of duration of long COVID throughout the manuscript.

355 It should say: “…whereas Germany only used national life tables”.

**Response :** It is updated as proposed (line 363).

356 -364

I would suggest to delete or change this. The case definition in France and Germany is quite similar what differs is the data source (surveillance data on notifiable diseases in Germany, CoD statistics in France). However, if you want to explain the huge differences in DALY I would suggest mainly the following points: (i) much more infections in France (ii) much more deaths in France (iii) use of GBD life tables in France. (i) and (ii) can in part be explained by the fact that the 2nd wave was more or less over by the end of 2020 in France but had its peak not before January 2021 in Germany.

**Response :** Thank you very much for this input. We have updated the text as suggested.

Discussion (line 356-364)

*« In France, YLL was higher (982 531) than in Germany (303 608) despite having a smaller population size. Both countries had different mortality data sources (i.e., surveillance data on notifiable diseases in Germany and causes of death statistics in France). France recorded a higher number of infections and deaths in 2020 than in Germany. The large difference in disease burden between these two countries could be explained by the fact that second wave was more or less over by the end of 2020 in France, whereas in Germany, the peak of infections was not reached before January 2021. Most importantly, France used the GBD reference life table, whereas Germany used national life expectancy tables to estimate YLL. »*

367 et seq

Anyway, the impact of YLD on DALY remains very limited and the issue if or how long COVID is considered cannot explain the main differences in DALY between countries. An explanation should always put the focus on the mortality part. This should be clearly stated somewhere.

**Response :** We agreed and have added the following text :

Discussion (line 366-372)

*“It is important to take into account, which post-acute consequences were considered when interpreting these results. All studies estimated a large contribution of YLL to DALYs, compared to YLD. Overall differences in estimated COVID-19 disease burdens across countries may reflect differences in disease surveillance systems, data collection systems and hospital care as well as the heterogeneity in impact of COVID-19 during the first waves of the pandemic in 2020.”*

See line 371 In fact the impact on population health is limited compared to mortality.

**Response:** We agreed that mortality has much strong impact than morbidity even after considering the long duration of COVID long.

We have deleted this phrase from the abstract and discussion sections.

**Reviewer #2:**

**Comment of first round:**

My major concern with this paper is the implausibly **low duration of long-COVID**, estimated from an analysis, which in my view does not adequately distinguish censored subjects from those with events or either of these from missing data. Symptom duration was defined, roughly, as time from diagnosis to the first one-week period where neither symptoms nor hospitalization were reported; and those subjects who reported being seriously ill and then stopped logging were excluded from the analysis. Given that fatigue and brain fog are among the most common long-COVID symptoms, it is likely that the sickest patients more-often stopped reporting. Precisely the data, which we would need to draw adequate conclusions, are missing.

**Response :** We fully agree with the low duration of long-COVID and integrated an additional sensitivity analysis with a five-fold increase in baseline duration used for long-COVID (i.e., 28*5 = 140 days). This has been added in the method, results and discussion sections as follows :

Method – Scenario analyses (line 238)

*« Therefore, we assumed two scenarios, one with threefold (i.e., 84 days) and another one with a fivefold (i.e., 140 days) increase in duration of post-acute consequences. The choice of 84 and 140 days was based on published literature (Moreno-Pérez et al, Huang et al) and experts’inputs . »*

Results- Scenario analyses (line 313)

*« Third, varying […] to 84 days and 140 days, it led to an increase of 188% […] and 375% with a total of 18 863 YLD, respectively. The duration of 140 days had the highest effect on DALYs (i.e., 1 001 394).*

*Fig 5 is updated accordingly.*

Discussion- Strength and limitations (line 400-405)

*« Fifth, we […], using a fivefold increase in duration of post-acute consequences (i.e., 140 days) […]. Nevertheless, the duration of post-acute consequences of COVID remains uncertain and more research is needed to draw conclusions on its duration. »*

Supporting information-S2 Table is updated accordingly.

**Comment of first round**

I would prefer if the current publication relied on studies, which actively contact patients, such as those reviewed in <https://www.nature.com/articles/s41591-021-01283-z>. These are likely to be much less biased by incomplete ascertainment, especially those such as Huang et al (cited in the current paper) with near-complete response; or Kim et al (<https://bmcinfectdis.biomedcentral.com/articles/10.1186/s12879-022-07062-6>). Even these are likely to underestimate duration somewhat, since post infectious conditions tend to relapse and remit: a patient who is recovered at the time of follow up may relapse again. This should be mentioned in the Discussion.

**Response**

We have already cited this review in the first round of revisions and have mentioned the underestimation of the duration of long COVID-19.

Now we updated the text and added a more detailed paragraph in the discussion section on what different studies have found in the context of long-COVID as follows :

Discussion – Implications for public health

Line 442-454

« *The current literature on post-acute COVID-19 syndrome based on clinical studies highlighted the organ-specific sequelae of COVID-19 survivors, especially the persistence of symptoms after discharge from the hospital at 60 – 120 days follow up (Nalbandiean et al). For example, the most common symptoms persisted beyond/after acute COVID-19 infection were fatigue (upto ~64% at 60 days followup)(Carfi et al, Haplin et al, Carvalho-Schneider et al, Arnold et al, Moreno-Pérez et al, Garrigues et al) , dyspnea (42-66% at 60-100 days followup) (Carfi et al, Chopra et al, Halpin et al, Garrigues et al), chest pain (upto ~ 20% at 60 days followup)(Carfi et al, Carvalho-Schneider et al) and sleep disturbance (24 -31% at 90-120 days followup)(Arnold et al, Garrigues et al). This evidence highlights the health care needs for patients with sequelae of COVID-19, such as fatigue, sleep disturbance, which are potentially not measurable in the data used (since they do not lead to hospital stays), and therefore, the burden of long-COVID is underestimated. These evidences suggest that developing integrated approaches across different disciplines for improved mental and physical health of survivors of COVID-19 in the long term are required (Nalbandiean A et al).”*

**Comments of first round:**

I also request that clinical manifestations other than long-COVID, such as myocarditis and psychiatric illness, be considered. These are described in Nalbandian et al, cited above. For simplicity and to avoid making the paper too long, I would be satisfied if their incidence rates were summed and the disability weights averaged to provide a single term for “all other conditions.”

**Response**

We have discussed this point with our team and still consider that this additional analysis creates difficulties due to the following reasons:

**First,** our estimates were calculated based on the consensus model developed by the European Burden of Disease experts (validated & published at [SSPH+ | Burden of Disease Methods: A Guide to Calculate COVID-19 Disability-Adjusted Life Years (ssph-journal.org)](https://www.ssph-journal.org/articles/10.3389/ijph.2021.619011/full)). This model does not take into account the “all other conditions” in the context of long COVID. This would require a consensus on defining the scope of long COVID in relation to the burden of disease calculations, in terms of what symptoms persisted after acute COVID-19 and the relevant disability weights to be used. Currently, there is no “averaged disability weight” that could take into account “all other conditions” proposed by the reviewer. While we are aware that the team that developed the consensus model, is working on updating the relevant parameters for long-COVID, at this stage it is not available yet. We have highlighted this point as a recommendation in the discussion and the main conclusion sections

Discussion (line 454)

*“Further research is needed to develop an updated consensus on defining the scope of long COVID in relation to the burden of disease calculations, especially in terms of what symptoms persisted after acute COVID-19 infections, their durations and the estimation of relevant disability weights.”*

Conclusions (line 470)

*“This is still a new domain for research and more evidence is needed to understand the dynamics of the post-acute consequences of this infection in terms of persistence of certain symptoms, their durations and relevant disability weights, in relation to burden of disease calculations.”*

**Second,** we think summing up the incidence rates of the post-acute COVID-19 studies reported in Nalbandian et al review (<https://www.nature.com/articles/s41591-021-01283-z>) (suggested by the reviewer 2) is not directly applicable to the French context. For example, these estimates are high by design. Studies included those patients who were hospitalized and were followed up, which arguably could increase the need for a control group (11% of these patients are still off on sick leave on day 60). An important methodological limitation of these clinical studies is the lack of a control group/comparator that may risk having biased estimates as well.

The persistence of different types of symptoms suffered by long COVID patients and their duration, are variable from person to person. This is very important and interesting aspect to be explored. As mentioned previously, the focus of this paper was to calculate the burden of COVID-19 using all national available data in France. We did not focus on post-acute COVID-19 survivors to characterise the impact of long COVID especially in context of “all other conditions”. This would require another method adopted to long COVID and using national available data. At time the study was conducted, some studies are ongoing to collect the relevant data.

Nevertheless, to account for this important comment, we have added a more detailed paragraph in the discussion section (implications for public health; line 442-454) describing what these studies have found in context of long-COVID.

**Third,** *« I also request that clinical manifestations other than long-COVID, such as myocarditis and psychiatric illness, be considered.* (Reviewer’s comment*) ».*

In the review by Nalbandian et al, a study by Rajpal S et al, included only 26 competitive college athletes with mild or asymptomatic SARS-CoV-2 infection, cardiac MRI revealed features diagnostic of myocarditis in 15% of participants, and previous myocardial injury in 30.8% of participants. This has a very small sample size. In table 1 of Nalbandian et al review, myocarditis was not reported as the most frequent and persisting symptom by different studies.

We fully agree that clinical manifestations other than long-COVID, such as myocarditis and psychiatric illness are very important to explore the organ-specific sequelae of COVID-19 survivors with persistent symptoms. It is not easy to take into account the myocarditis and psychiatric illness in the context of long COVID and we did not perform this analysis due to two main reasons (as mentioned above) : first, we think it would require a specific method adopted to long COVID and second, lack of relevant data collected from local data sources to explore this aspect. Moreover, long COVID is a new domain of research and it would need more longitudinal studies to draw conclusions on this point.

**Comments of first round:**

Lastly, I request an extension of the literature review, comparing the current estimates with those others have found. The current paper may be the first to consider both in-hospital morbidity and long-COVID, in addition to death (many others explicitly exclude long-COVID, e.g. <https://www.researchsquare.com/article/rs-1026794/v2>) however it estimates a much higher share of YLD than <https://www.jclinepi.com/article/S0895-4356(21)00339-5/fulltext> . Please compare your findings to those of the latter paper, as well as any others you may find that compute DALY for long-COVID.

**Response**

Just a remark that we have cited relevant articles and compared the findings with those that applied the same disability weight and same disease model.

The latter paper (<https://www.jclinepi.com/article/S0895-4356(21)00339-5/fulltext> ) has applied disability weights used for chronic fatigue syndrome (CFS) for long COVID and has applied a different model than the European Burden o Disease consensus model. One of the main limitations of that study was the use of disability weights for CFS and long COVID has symptoms that CFS does not, leading to underestimation of the true disability weight of long COVID.

**Comments of second round:**

Authors did not implement the additional analyses I requested: instead they added a few sentences saying that they hadn't done so. They easily could have done those analyses with existing data.
Without those additional analyses the paper substantially misrepresents the situation and has potential to mislead policy if published as it stands. It could most charitably be described as incomplete / biased.

Please make the changes I requested in the previous round of edits, especially the sensitivity analysis using numbers for long-COVID prevalence obtained from contacting patients.

**Response:** We agree that we did not fully perform the additional analyses as you suggested. Nevertheless, we have integrated an additional sensitivity analysis by increasing the duration of long-COVID to 140 days in the main results that highlighted the impact of duration of long COVID on YLD estimate, which was rather easy.

As mentioned above, we fully agree with all your comments related to the persistence of different symptoms and the clinical manifestations other than long COVID among COVID survivors. Indeed, these are very important aspects in the context of long COVID. It would require another study with a specific method adopted to characterise the impact of long COVID, using local data sources, reflecting the French context.
