# Supplementary material for: Direct impact of COVID-19 by estimating disability-adjusted life years at national level in France in 2020
Source: PLoS One. 2023 Jan 24;18(1):e0280990. doi: 10.1371/journal.pone.0280990 (PMC9873186; doi:10.1371/journal.pone.0280990)
Supplement: Attachment — Submitted filename: Dear Editor.docx [file pone.0280990.s007.docx]

Dear Editor,

Thank you for the opportunity to review this paper. My major concern is the assumption that long-COVID duration never exceeds 140 days, an assumption which is already disproven. However, the this assumption is based on an external source (European Burden of Disease Network consensus disease model) which is likely to be widely used, and this the paper fills a needed gap in the literature.

I request that this assumption be highlighted in Abstract and perhaps also in Discussion, as follows:

**Abstract:**

Background The World Health Organization declared a pandemic of coronavirus disease 2019 (COVID-19), caused by severe acute respiratory syndrome coronavirus 2 (SARS-CoV2), on March 11, 2020. The standardized approach of disability-adjusted life years (DALYs) allows for quantifying the combined impact of morbidity and mortality of diseases and injuries. The main objective of this study was to estimate the direct impact of COVID-19 in France in 2020, using DALYs to combine the population health impact of infection fatalities, acute symptomatic infections and their post-acute consequences in the 140 days following the initial infection.

Methods National mortality, COVID-19 screening, and hospital admission data were used to calculate DALYs based on the European Burden of Disease Network consensus disease model. Scenario analyses were performed by varying the number of symptomatic cases and duration of symptoms up to a maximum of 140 days, defining COVID-19 deaths using the underlying, and associated, cause of death.

Results In 2020, the estimated DALYs due to COVID-19 in France were 990 710 (1472 per 100 000), with 99% of burden due to mortality (982 531 years of life lost, YLL) and 1% due to morbidity (8179 years lived with disability, YLD) in the 140 days following infection. The contribution of YLD due to acute symptomatic infections among people younger than 70 years was higher (67%) than among people aged 70 years and above (33%). Post-acute consequences contributed to 49% of the total morbidity burden. YLL among people aged 70 years and above, contributed to 74% of the total YLL. Conclusions COVID-19 had a substantial impact on population health in France in 2020. The majority of population health loss was due to mortality. Men had higher population health loss due to COVID-19 than women. Post-acute consequences of COVID-19 had a large contribution to the YLD component of the disease burden, even in the scenario where long-COVID duration is limited to 140 days. Further research is recommended to assess the impact of health inequalities associated with these estimates.

**Discussion, first paragraph:**

Our study is the first to estimate DALYs associated with the direct health impact of COVID 19 in France in 2020, the first year of the pandemic. When long-COVID duration was capped at a maximum of 140 days, the majority of population health loss was due to mortality, which contributed to 99% to the estimated DALY. People aged 70 years and 16 above had higher health loss due to mortality when compared to people aged younger than 70 years. On the contrary, people aged younger than 70 years had higher disability due to acute COVID-19 infections than those aged 70 years and above. Our analysis highlighted that even if long-COVID resolves after less than six months, the post-acute consequences of COVID-19 had a large contribution to the YLD component of disease burden. Moreover, we observed that women had higher YLD due to the post-acute consequences of COVID-19 than men (2147 vs 1824). Other studies have also highlighted that females were more likely to have post-COVID syndrome than males [26-28]. However, population health loss due to mortality and morbidity due to COVID-19 infections, was higher among men than women.

**Discussion, line 400**

Fifth, the maximum duration of long-COVID is currently unknowable but is known to be at least several years. Its duration is a major determinant of YLD of post-acute consequences of COVID-19. Although our source assumes a maximum duration of 28 days, we used ~~using~~ a fivefold increase in duration of post-acute consequences (i.e., 140 days) as a scenario analysis, to evaluate the impact of a much longer duration of post- acute consequences of COVID on YLD estimates. Nevertheless, the duration of post-acute consequences of COVID-19 remains uncertain and more research is needed to draw conclusions on its duration.
