# Supplementary material for: Direct impact of COVID-19 by estimating disability-adjusted life years at national level in France in 2020
Source: PLoS One. 2023 Jan 24;18(1):e0280990. doi: 10.1371/journal.pone.0280990 (PMC9873186; doi:10.1371/journal.pone.0280990)
Supplement: Attachment — Submitted filename: Response to reviewers _R3.docx [file pone.0280990.s008.docx]

Paris, December 1, 2022

Subject: Major revisions for “PONE-D-22-11149: Direct impact of COVID-19 by estimating disability-adjusted life years at national level in France in 2020”

Dear Dr. Mossong,

We would like to thank you again for considering our manuscript.

We have modified the manuscript according to the suggestions of reviewer 2. Nevertheless, we deeply appreciate that both reviewers’ gave useful comments that have improved the quality of our manuscript.

In response to the reviewer 2 suggestion, we would like to highlight this point that we used the baseline duration of long-COVID as 28 days. Our main results of YLD (i.e., 1% contribution of YLD: 8179) included both types of infections: acute infections (i.e., mild, moderate and severe) and long-COVID (i.e., with baseline duration of 28 days). The duration of 140 days for long-COVID is part of one of the sensitivity analyses. Therefore, we updated the text in the abstract and in the discussion sections as suggested by the reviewer 2, where it was appropriate.

There are two manuscript files (one with track changes and one clean version). The line numbers in the comment file correspond to the clean version file.

We deeply appreciate your kind support for the publication of this manuscript.

Yours sincerely,

Romana Haneef

**Response to reviewers’ comments**

**Reviewer #2:** Thank you for the opportunity to review this paper. My major concern is the assumption that long-COVID duration never exceeds 140 days, an assumption which is already disproven. However, this assumption is based on an external source (European Burden of Disease Network consensus disease model) which is likely to be widely used, and this the paper fills a needed gap in the literature.

I request that this assumption be highlighted (in red) in Abstract and perhaps also in Discussion, as follows:

**Response:** Thank you very much for these suggestions and your kind support.

As discussed in the previous round of revisions and mentioned in the discussion section, the duration of long-COVID is uncertain due to lack of precise data that could help to conclude the real duration of long-COVID. Therefore, to take into account the uncertainty around this duration (already mentioned in the method section-line 237-240); we applied two sensitivity analyses for duration of long-COVID of 84 days and 140 days. Over time, when more evidence will be available, then we can hope to approach a more accurate duration of long-COVID. Nevertheless, these sensitivity analyses help to evaluate the impact of the duration on YLD estimates.

Text in red: suggestions by the reviewer

Text in black, **bold**, *italic* font: Authors’ response

We have highlighted the assumptions in the abstract as well as in the discussions sections as follows suggested by the reviewer:

**Abstract:**

1. **Background:** The World Health Organization […]. The main objective of this study was to estimate the direct impact of COVID-19 in France in 2020, using DALYs to combine the population health impact of infection fatalities, acute symptomatic infections and their post-acute consequences in the 140 days following the initial infection.

**Response:** We have updated the text as follows because our main results of YLD (i.e., 1% contribution of YLD: 8179) included both types of infections: acute infections (i.e., mild, moderate and severe) and long-COVID (i.e., with baseline duration of 28 days up to 140 days), following the initial infection.

***« Background****: The World Health Organization […]. The main objective of this study was to estimate the direct impact of COVID-19 in France in 2020, using DALYs to combine the population health impact of infection fatalities, acute symptomatic infections and their post-acute consequences,* ***in 28 days (baseline) up to 140 days,*** ***following the initial infection. »***

2. **Methods**: National mortality, COVID-19 screening, and hospital admission data were used to calculate DALYs based on the European Burden of Disease Network consensus disease model. Scenario analyses were performed by varying the number of symptomatic cases and duration of symptoms up to a maximum of 140 days, defining COVID-19 deaths using the underlying, and associated, cause of death.

**Response:** The text is updated as suggested.

***« Methods:*** *[….]. Scenario analyses were performed by varying the number of symptomatic cases and duration of symptoms* ***up to a maximum of 140 days****, defining COVID-19 deaths using the underlying, and associated, cause of death. »*

3. **Results:** In 2020, the estimated DALYs due to COVID-19 in France were 990 710 (1472 per 100 000), with 99% of burden due to mortality (982 531 years of life lost, YLL) and 1% due to morbidity (8179 years lived with disability, YLD) in the 140 days following infection.

**Response:** As mentioned earlier, our baseline duration of long-COVID was 28 days according to our disease model. Our main results of YLD (i.e., 1% contribution of YLD: 8179) included both types of infections: acute infections (i.e., mild, moderate and severe) and long-COVID (i.e., with baseline duration of 28 days). The duration of 140 days for long-COVID is part of one of the sensitivity analyses. We updated the text as follows:

*« In 2020, the estimated DALYs due to COVID-19 in France were 990 710 (1472 per 100 000), with 99% of burden due to mortality (982 531 years of life lost, YLL) and 1% due to morbidity (8179 years lived with disability, YLD),* ***following the initial infection. The contribution of YLD reached 375%, assuming the duration of 140 days of post-acute consequences of COVID-19. »***

4. **Conclusions:** COVID-19 had a substantial impact on population health in France in 2020. The majority of population health loss was due to mortality. Men had higher population health loss due to COVID-19 than women. Post-acute consequences of COVID-19 had a large contribution to the YLD component of the disease burden, even in the scenario where long-COVID duration is limited to 140 days. Further research is recommended to assess the impact of health inequalities associated with these estimates.

**Response:** As mentioned earlier, our baseline duration of long-COVID is 28 days, even with this shortest duration, the long-COVID burden is large and with 140 days (part of sensitivity analysis), long-COVID burden was increased by 375%. The text is updated accordingly.

***« Conclusions: [….].*** *Post-acute consequences of COVID-19 had a large contribution to the YLD component of the disease burden****, even when we assume the shortest duration of 28 days, long COVID burden is large.*** *Further research is recommended to assess the impact of health inequalities associated with these estimates. »*

**Discussion, first paragraph:**

5. Our study is the first to estimate DALYs associated with the direct health impact of COVID 19 in France in 2020, the first year of the pandemic. When long-COVID duration was capped at a maximum of 140 days, the majority of population health loss was due to mortality, which contributed to 99% to the estimated DALY.

**Response**: As mentioned above, the main results of YLD (8179) are calculated based on acute infections (i.e., mild, moderated and severe) and long-COVID (with a baseline duration of 28 days). We updated the text as follows (line 334):

*“The majority of population health loss was due to mortality, which contributed to 99% to the estimated DALY.* ***This finding is in the context of long COVID being capped at a maximum of 140 days, but would still hold if long COVID duration was uncapped for the entire year.”***

6. Our analysis highlighted that even if long-COVID resolves after less than six months, the post-acute consequences of COVID-19 had a large contribution to the YLD component of disease burden.

**Response:** We have updated the text as suggested (line 340).

*Our analysis highlighted that* ***even if long COVID resolves after less than six months,*** *the post-acute consequences of COVID-19 had a large contribution to the YLD component of disease burden.*

**Discussion, line 400**

7. Fifth, the maximum duration of long-COVID is currently unknowable but is known to be at least several years. Its duration is a major determinant of YLD of post-acute consequences of COVID-19. Although our source assumes a maximum duration of 28 days, we used a fivefold increase in duration of post-acute consequences (i.e., 140 days) as a scenario analysis, to evaluate the impact of a much longer duration of post- acute consequences of COVID on YLD estimates.

**Response**: Thanks for these inputs. We have updated the text as follow (line 406):

*Fifth,* ***the maximum duration of long COVID is currently unknown but a subset of patients could suffer these consequences through the entire annual period of study and we still lack the data to assess these consequences. The duration of long COVID is a major determinant of*** *YLD of post-acute consequences of COVID-19.* ***Our disease model was based on a duration of 28 days, we used*** *a threefold and fivefold (i.e., 84 days and 140 days) increase in duration of post-acute consequences, as sensitivity analysis, to evaluate the impact of a much longer duration of post- acute consequences of COVID on YLD estimates.*
